# *CRLF2* rearrangement in Ph-like acute lymphoblastic leukemia predicts relative glucocorticoid resistance that is overcome with MEK or Akt inhibition

**DOI:** 10.1371/journal.pone.0220026

**Published:** 2019-07-18

**Authors:** Lauren K. Meyer, Cristina Delgado-Martin, Shannon L. Maude, Kevin M. Shannon, David T. Teachey, Michelle L. Hermiston

**Affiliations:** 1 Department of Pediatrics, University of California, San Francisco, CA, United States of America; 2 Department of Pediatrics, University of Pennsylvania, Philadelphia, PA, United States of America; European Institute of Oncology, ITALY

## Abstract

Philadelphia chromosome-like (Ph-like) acute lymphoblastic leukemia (ALL) is a genetically heterogeneous subtype of B-cell ALL characterized by chromosomal rearrangements and mutations that result in aberrant cytokine receptor and kinase signaling. In particular, chromosomal rearrangements resulting in the overexpression of cytokine receptor-like factor 2 (*CRLF2*) occur in 50% of Ph-like ALL cases. *CRLF2* overexpression is associated with particularly poor clinical outcomes, though the molecular basis for this is currently unknown. Glucocorticoids (GCs) are integral to the treatment of ALL and GC resistance at diagnosis is an important negative prognostic factor. Given the importance of GCs in ALL therapy and the poor outcomes for patients with *CRLF2* overexpression, we hypothesized that the aberrant signal transduction associated with *CRLF2* overexpression might mediate intrinsic GC insensitivity. To test this hypothesis, we exposed Ph-like ALL cells from patient-derived xenografts to GCs and found that *CRLF2* rearranged (*CRLF2*_*R*_) leukemias uniformly demonstrated reduced GC sensitivity *in vitro*. Furthermore, targeted inhibition of signal transduction with the MEK inhibitor trametinib and the Akt inhibitor MK2206, but not the JAK inhibitor ruxolitinib, was sufficient to augment GC sensitivity. These data suggest that suboptimal GC responses may in part underlie the poor clinical outcomes for patients with *CRLF2* overexpression and provide rationale for combination therapy involving GCs and signal transduction inhibitors as a means of enhancing GC efficacy.

## Introduction

Philadelphia chromosome-like (Ph-like) acute lymphoblastic leukemia (ALL) is a subtype of B-cell ALL that displays a gene expression profile resembling that of *BCR-ABL1*-positive ALL but lacks the *BCR-ABL1* translocation. Instead, Ph-like ALL is characterized by genetic alterations that result in aberrant cytokine receptor and kinase signaling [1]. In particular, 50% of Ph-like ALL cases harbor chromosomal rearrangements that result in overexpression of cytokine receptor-like factor 2 (*CRLF2*), which heterodimerizes with the interleukin-7 receptor alpha chain to form the thymic stromal lymphopoietin receptor (TSLPR) [2]. Upon activation by TSLP ligand, TSLPR signals through the JAK/STAT, Ras/MAPK, and PI3K/Akt/mTOR pathways [3]. Importantly, overexpression of *CRLF2* is associated with a particularly poor prognosis, with these patients demonstrating significantly worse relapse-free survival relative to patients without *CRLF2* overexpression [2,4]. While the molecular basis for this clinical observation is currently unknown, this suggests that these patients may have intrinsic resistance to conventional chemotherapy.

Glucocorticoids (GCs) are an integral component of therapy for patients with ALL [5]. GCs act by binding to a cytoplasmic GC receptor (GR), which promotes translocation of the GC/GR complex to the nucleus and induction of a transcriptional program that results in apoptosis in lymphoid cells [6]. Downstream effectors of signal transduction pathways have been shown to inhibit several of these processes, including nuclear translocation of ligand-activated GR [7,8] and induction of the GR transcriptional program [9]. Furthermore, cytokines present in the microenvironment have been shown to promote GC resistance in lymphoblasts by activating these signal transduction pathways [10–12].

Given the importance of GC resistance in ALL and the comparatively poor clinical outcomes for patients with *CRLF2* overexpression, we hypothesized that the aberrant signal transduction associated with *CRLF2* overexpression might contribute to suboptimal responses to GC therapy. We tested this hypothesis by assessing *in vitro* drug responses in *CRLF2-*rearranged (*CRLF2*_*R*_) and -non-rearranged (*CRLF2*_*NR*_) cells from patient-derived xenografts of Ph-like ALL, and found that *CRLF2*_*R*_ samples were uniformly less sensitive to GCs relative to many *CRLF2*_*NR*_ samples. Furthermore, we demonstrated that GC sensitivity could be significantly enhanced with concomitant signal transduction inhibition, providing rationale for further evaluation of a combination therapy strategy as a means of augmenting GC sensitivity in patients with *CRLF2* overexpression.

## Materials and methods

To assess GC sensitivity in Ph-like ALL cells, cryopreserved splenocytes were obtained from 19 patient-derived xenografts of high-risk Ph-like ALL established from treatment-naïve diagnostic samples banked in the Children’s Hospital of Philadelphia leukemia biorepositories. Written informed consent for the use of diagnostic specimens for future research was obtained from patients or their guardians at the time of sample collection, according to the Declaration of Helsinki and the National Cancer Institute and the institutional review board of Children’s Hospital of Philadelphia, which approved this study. As a control to assess for *in vitro* drug toxicity, peripheral blood mononuclear cells (PBMCs) from healthy donors were obtained from Vitalant. Rearrangements involving the *CRLF2* locus were detected as previously described [2]. Cells were thawed, allowed to rest for 1 hour at 37°C, then cultured at 37°C in media supplemented with 25 ng/mL recombinant human TSLP (Peprotech, Rocky Hill, NJ, USA). Ruxolitinib (Selleckchem, Houston, TX, USA), a JAK1/2 inhibitor, was used at 500nM while dexamethasone (DEX; Sigma-Aldrich, St. Louis, MO, USA), a synthetic GC, trametinib (Selleckchem), a MEK1/2 inhibitor, and MK2206 (Selleckchem), a pan-Akt inhibitor, were used at 1μM. Cells were harvested at 48 hours and flow cytometry was performed as previously described using a FACSVerse flow cytometer (BD Biosciences, San Jose, CA, USA) [13]. Antisera included anti-human CD45, anti-cleaved caspase-3, anti-TSLPR, anti-STAT5 (pY694), anti-ERK1/2 (pT202/pY204), anti-Akt (pS473) (BD Biosciences), and anti-GR (Cell Signaling, Danvers, MA, USA). Signal strength was quantified as the median fluorescence intensity (MFI) of cells negative for cleaved caspase-3 within the human CD45-positive gate. Viability assays were performed via calculation of the frequency of Hoechst (ThermoFisher, Waltham, MA, USA) negative cells in the human CD45-positive gate. All viability data are presented as the percentage of viable cells in the drug-treated condition relative to the percentage of viable cells in the corresponding vehicle control condition for each individual sample. The average viability across all samples in the vehicle control condition at 48 hours was 39% +/- 23%. Statistical analyses were performed using Prism 8 (GraphPad, San Diego, CA, USA). All tests were two-sided and the threshold for significance was p≤0.05. Specifically, comparisons between groups were made using t-tests, with one-way ANOVA and Tukey’s method for multiple comparisons adjustment used for comparisons of three or more groups. Drug-drug interactions were assessed using the Bliss independence model of synergy [14]

## Results

Eleven of the 19 Ph-like leukemias were positive for *CRLF2* rearrangements. Of these, nine expressed transcripts corresponding to the *IGH@-CRLF2* rearrangement and two were positive for the *P2RY8-CRLF2* rearrangement. Seven of these *CRLF2*_*R*_ leukemias had concomitant activating mutations in *JAK2*. All eight *CRLF2*_*NR*_ Ph-like leukemias had point mutations or rearrangements involving other kinases previously implicated in Ph-like ALL [15] (S1 Table).

**Fig 1:**
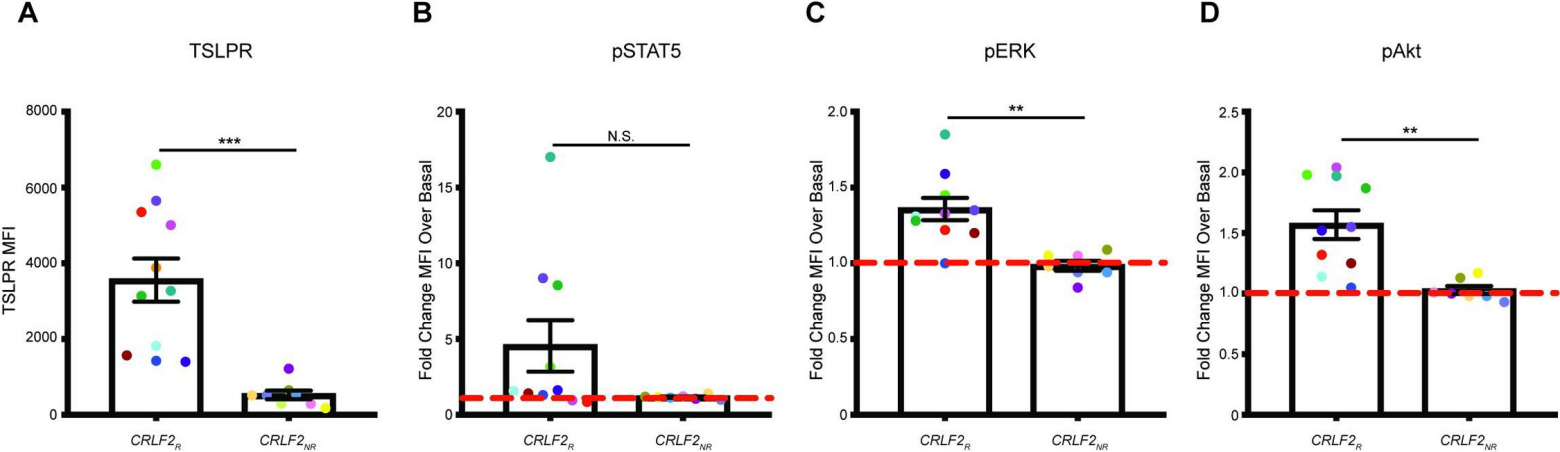
*CRLF2*_*R*_ leukemias demonstrate TSLPR overexpression and increased signal transduction activity relative to *CRLF2*_*NR*_ leukemias. (**A**) MFI of TSLPR cell surface protein expression as determined by antibody staining and flow cytometry in cells from *CRLF2*_*R*_ and *CRLF2*_*NR*_ samples. Individual patient samples are shown in the same color in all figures and in S1 Table. (**B-D**) Induction of (**B**) pSTAT5, (**C**) pERK, and (**D**) pAkt in *CRLF2*_*R*_ and *CRLF2*_*NR*_ samples in response to a 30-minute stimulation with 25ng/mL TSLP, presented as the fold change in MFI following TSLP stimulation relative to the basal condition. Samples JH721 and NL432 were not included in this analysis due to limitations in cell number. Error bars represent the standard error of the mean. Statistical significance was assessed using a two-sample t-test. ****p<0.0001, ***p<0.001, **p<0.01, *p<0.05.

Both *IGH@-CRLF2* and *P2RY8-CRLF2* translocations have been reported to result in *CRLF2* overexpression [16]. Consistent with these data, the *CRLF2*_*R*_ leukemias analyzed in the current study exhibited higher cell surface TSLPR protein expression relative to *CRLF2*_*NR*_ leukemias (p = 0.0003; Fig 1A). We next assessed the induction of phosphorylated (p) STAT5, ERK, and Akt in response to short-term TSLP stimulation in *CRLF2*_*R*_ and *CRLF2*_*NR*_ leukemias. Four of 10 *CRLF2*_*R*_ leukemias, including two that lacked a concomitant *JAK2* mutation, markedly induced pSTAT5 in response to short-term TSLP stimulation, while *CRLF2*_*NR*_ leukemias did not (Fig 1B). *CRLF2*_*R*_ leukemias also induced pERK and pAkt in response to TSLP, with no induction in the *CRLF2*_*NR*_ leukemias (p = 0.001 and p = 0.002 versus *CRLF2*_*NR*_ leukemias, respectively; Fig 1C and 1D). There were no differences in basal levels of pSTAT5, pERK, or pAkt between *CRLF2*_*R*_ and *CRLF2*_*NR*_ leukemias (S1 Fig).

**Fig 2:**
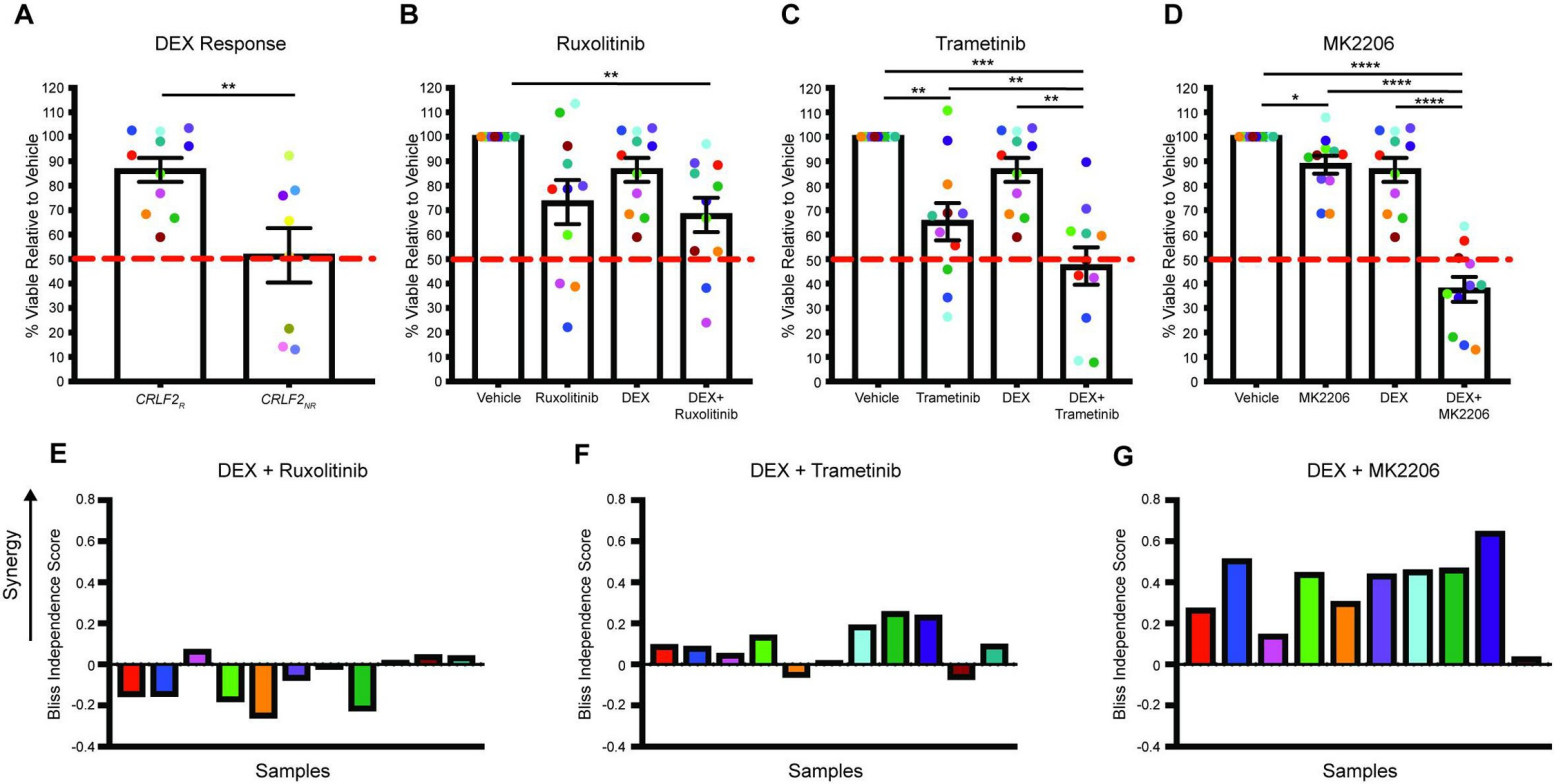
*CRLF2*_*R*_ leukemias are uniformly DEX resistant and can be sensitized to DEX with targeted inhibition of MEK or Akt. (**A**) Cell viability in response to 1μM DEX, presented as the percentage of viable cells in the DEX treated condition relative to the vehicle control condition in *CRLF2*_*R*_ and *CRLF2*_*NR*_ samples. (**B-D**) Cell viability of *CRLF2*_*R*_ samples following exposure to 1μM DEX, (**B**) 500nM ruxolitinib, (**C**) 1μM trametinib, (**D**) 1μM MK2206, or a combination of DEX and a targeted inhibitor presented as the percentage of viable cells in the drug treated conditions relative to the vehicle control condition. **(E-G)** Bliss independence scores for the combination of DEX and ruxolitinib **(E)**, trametinib **(F)**, or MK2206 **(G)** in *CRLF2*_*R*_ samples, in which positive values indicate a synergistic interaction between the two agents. Error bars represent the standard error of the mean. Statistical significance was assessed using a two-sample t-test (A) or one-way ANOVA with Tukey’s method for multiple comparisons adjustment (B-D). ****p<0.0001, ***p<0.001, **p<0.01, *p<0.05.

To test the hypothesis that aberrant signal transduction might impair GC sensitivity in *CRLF2*_*R*_ leukemias, we assessed the response to DEX *in vitro*. In the presence of DEX, all 11 *CRLF2*_*R*_ leukemias retained greater than 50% survival at 48 hours, in contrast to only four *CRLF2*_*NR*_ leukemias (p = 0.006 versus *CRLF2*_*NR*_ leukemias; Fig 2A). Importantly, this difference in DEX sensitivity was not explained by differences in GR expression (S2A Fig). Given the uniform DEX insensitivity in the *CRLF2*_*R*_ leukemias, we asked whether inhibition of one or more signal transduction pathways downstream of TSLPR might augment DEX-induced cell death in these samples. We treated these leukemias with the combination of DEX and inhibitors of JAK1/2 (ruxolitinib), MEK (trametinib), or Akt (MK2206) at concentrations that attenuated TSLP-induced signaling in the *CRLF2*_*R*_ Ph-like ALL cell line Mutz-5 (S2B–S2D Fig) and that did not demonstrate toxicity in healthy PBMCs (S2E Fig). In the *CRLF2*_*R*_ samples, ruxolitinib had no significant single agent effect when assessed across the entire cohort, but it showed increased efficacy in samples with a *JAK2* mutation relative to those without (p = 0.03; S3A Fig). Furthermore, the combination of DEX and ruxolitinib was not more effective than either agent alone (Fig 2B). In contrast, trametinib and MK2206, which showed moderate single-agent activity (p = 0.005 and p = 0.04 versus vehicle, respectively), were significantly more effective in combination with DEX relative to either single agent alone (p = 0.005 and p<0.0001 versus DEX alone, respectively; Fig 2C and 2D). Bliss independence analysis confirmed these findings, demonstrating no interaction between DEX and RUX but cooperativity between DEX and trametinib and DEX and MK2206 (Fig 2E–2G). In the *CRLF2*_*NR*_ samples, these signal transduction inhibitors demonstrated variable effects, with some modulation of DEX sensitivity in several samples that demonstrated relative insensitivity to DEX as a single agent (S3B–S3D Fig).

## Discussion

Taken together, these data demonstrate that Ph-like ALL samples with *CRLF2* rearrangements uniformly demonstrate limited GC sensitivity *in vitro*. Furthermore, these data suggest a model whereby dysregulated signal transduction in the context of *CRLF2* overexpression contributes to relative GC resistance. This provides rationale for a clinical strategy involving combination therapy with GCs and signal transduction inhibitors as a means of enhancing GC efficacy. A precedent for this type of combination therapy approach includes the addition of ABL kinase inhibitors to standard chemotherapy regimens for children with Ph-positive ALL, which has dramatically improved clinical outcomes for these patients [17]. Together with the known prognostic value of GC sensitivity, our findings suggest that the inferior survival rates for patients with *CRLF2* overexpression may be driven at least in part by suboptimal responses to GC therapy_._ These data support further evaluation of *CRLF2* rearrangement status as a clinical biomarker for relatively poor sensitivity to GCs, thereby enabling the identification of patients who may benefit from the addition of molecularly targeted therapy to conventional anti-leukemia chemotherapy.

## Supporting information

S1 Fig MFI of basal (A) pSTAT5, (B) pERK, and (C) pAkt in CRLF2R and CRLF2NR leukemias. Error bars represent the standard error of the mean. Statistical significance was assessed using a two-sample t-test.
(TIF)

S2 Fig (A) MFI of intracellular GR protein expression as determined by flow cytometry in CRLF2R and CRLF2NR samples. (B-D) Histograms indicating levels of (B) pSTAT5, (C) pERK, or (D) pAkt following TSLP stimulation with or without one hour pre-treatment with 500nM ruxolitinib, 1μM trametinib, or 1μM MK2206, respectively, in the CRLF2R Ph-like ALL cell line Mutz-5. (E) Viability of PBMCs from three healthy donors following exposure to 500nM ruxolitinib, 1μM trametinib, or 1μM MK2206. Error bars represent the standard error of the mean. Statistical significance was assessed using a two-sample t-test (A) or one-way ANOVA with Tukey’s method for multiple comparisons adjustment (E).
(TIF)

S3 Fig (A) Cell viability of CRLF2R samples with JAK2 mutations versus those without JAK2 mutations following exposure to 500nM ruxolitinib. (B-D) Cell viability of CRLF2NR samples following exposure to 1μM DEX, (B) 500nM ruxolitinib, (C) 1μM trametinib, (D) 1μM MK2206, or a combination of DEX and a targeted inhibitor presented as the percentage of viable cells in the drug treated conditions relative to the vehicle control condition. Error bars represent the standard error of the mean. Statistical significance was assessed using a paired t-test (A) or one-way ANOVA with Tukey’s method for multiple comparisons adjustment (B-D). ****p<0.0001, ***p<0.001, **p<0.01, *p<0.05.
(TIF)

S1 Table Characteristics of Ph-like ALL samples. (TIF)
