# Supplementary figures and images for: CRLF2 rearrangement in Ph-like acute lymphoblastic leukemia predicts relative glucocorticoid resistance that is overcome with MEK or Akt inhibition
Source: PLoS One. 2019 Jul 18;14(7):e0220026. doi: 10.1371/journal.pone.0220026 (PMC6638974; doi:10.1371/journal.pone.0220026)

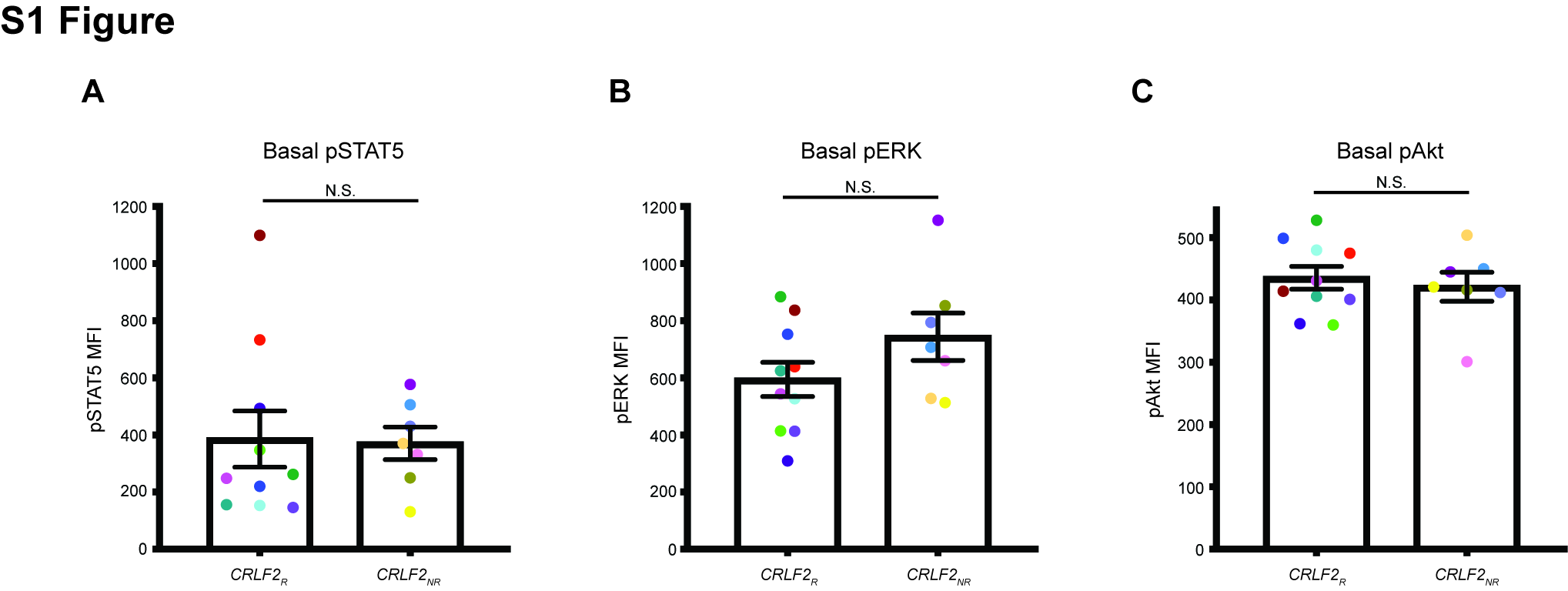

Supplement: S1 Fig — MFI of basal (A) pSTAT5, (B) pERK, and (C) pAkt in CRLF2R and CRLF2NR leukemias. Error bars represent the standard error of the mean. Statistical significance was assessed using a two-sample t-test. (TIF) [file pone.0220026.s001.tif]

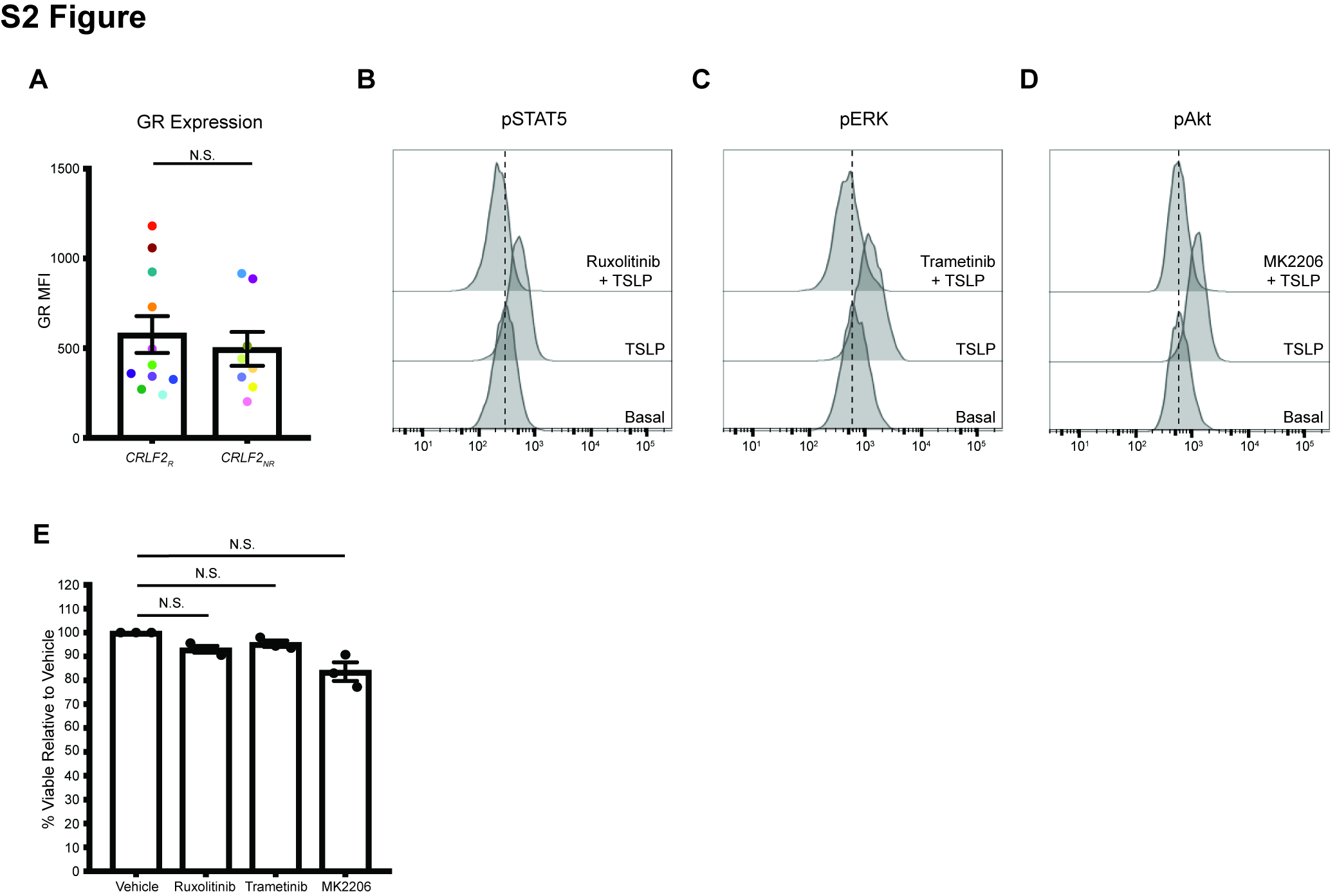

Supplement: S2 Fig — (A) MFI of intracellular GR protein expression as determined by flow cytometry in CRLF2R and CRLF2NR samples. (B-D) Histograms indicating levels of (B) pSTAT5, (C) pERK, or (D) pAkt following TSLP stimulation with or without one hour pre-treatment with 500nM ruxolitinib, 1μM trametinib, or 1μM MK2206, respectively, in the CRLF2R Ph-like ALL cell line Mutz-5. (E) Viability of PBMCs from three healthy donors following exposure to 500nM ruxolitinib, 1μM trametinib, or 1μM MK2206. Error bars represent the standard error of the mean. Statistical significance was assessed using a two-sample t-test (A) or one-way ANOVA with Tukey’s method for multiple comparisons adjustment (E). (TIF) [file pone.0220026.s002.tif]

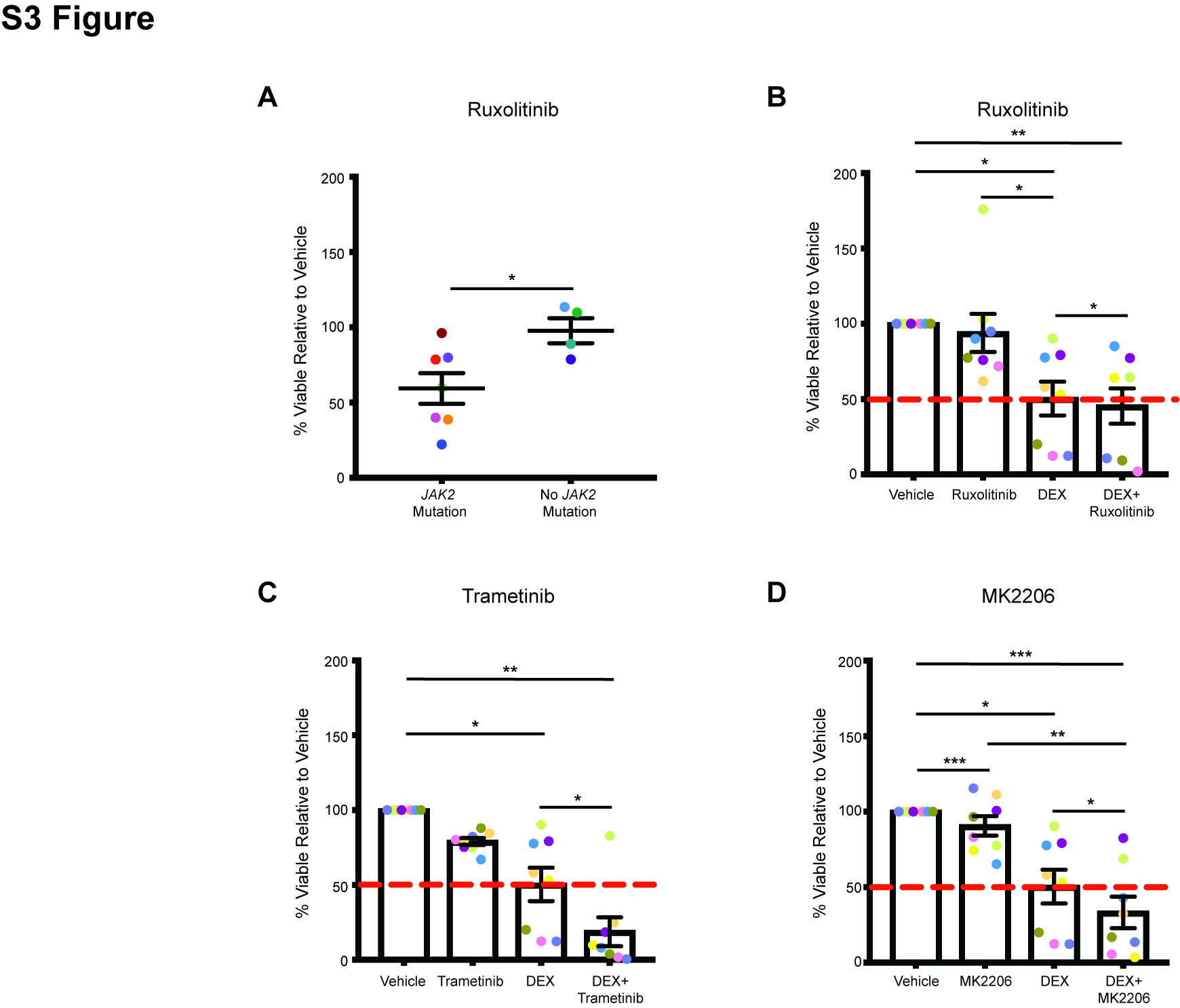

Supplement: S3 Fig — (A) Cell viability of CRLF2R samples with JAK2 mutations versus those without JAK2 mutations following exposure to 500nM ruxolitinib. (B-D) Cell viability of CRLF2NR samples following exposure to 1μM DEX, (B) 500nM ruxolitinib, (C) 1μM trametinib, (D) 1μM MK2206, or a combination of DEX and a targeted inhibitor presented as the percentage of viable cells in the drug treated conditions relative to the vehicle control condition. Error bars represent the standard error of the mean. Statistical significance was assessed using a paired t-test (A) or one-way ANOVA with Tukey’s method for multiple comparisons adjustment (B-D). ****p<0.0001, ***p<0.001, **p<0.01, *p<0.05. (TIF) [file pone.0220026.s003.tif]

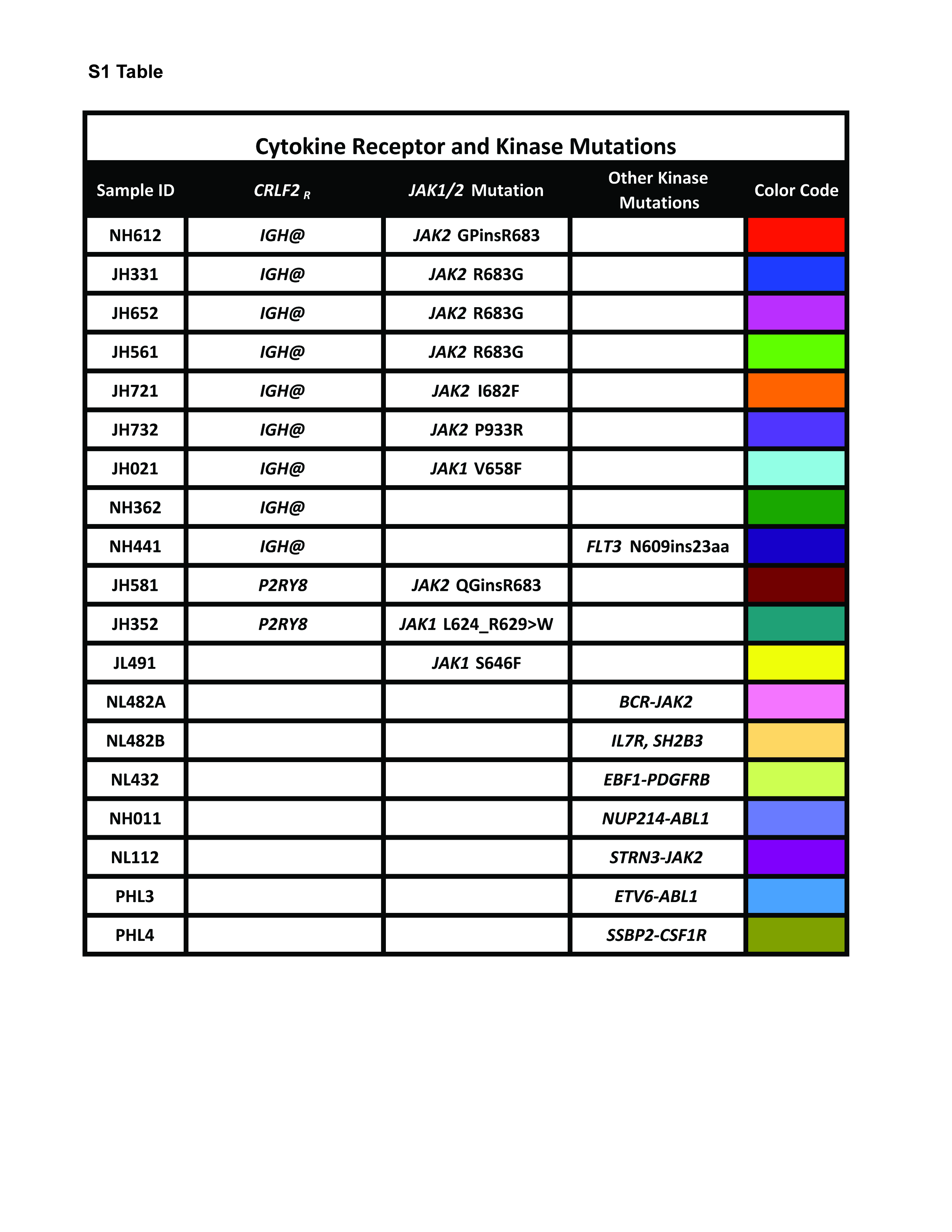

Supplement: S1 Table — (TIF) [file pone.0220026.s004.tif]
